# Aggressive fibromatosis in the infratemporal fossa presenting as trismus: a case report

**DOI:** 10.1186/s13256-018-1577-3

**Published:** 2018-02-19

**Authors:** Sunil Munakomi

**Affiliations:** Nobel Teaching Hospital, Kachanbari, Biratnagar, Nepal

**Keywords:** Trismus, Aggressive, Fibromatosis

## Abstract

**Background:**

Here we report a very rare entity of an infratemporal region aggressive fibromatosis in a 23-year-old Tharu man who had presented with the symptoms of painless but progressive trismus.

**Case presentation:**

We describe a case of aggressive fibromatosis in a 23-year-old Tharu man. Radiological imaging as well as an immunohistochemistry panel from a biopsy indicated a diagnosis of an aggressive fibromatosis. Since there was no aggravation in his trismus following surgery and because of his poor socioeconomic status, he was advised to attend regular follow-up visits without any adjuvant therapy.

**Conclusions:**

This case report adds to the notion of keeping the differential diagnosis of an aggressive fibromatosis in all patients presenting with progressive but painless trismus. The characteristic imaging findings as well an immunohistochemistry panel will help us clinch the correct diagnosis.

## Background

Aggressive fibromatosis (AF) is a very rare entity [1]. Its origin in the infratemporal region presenting solely as trismus is even rarer with only a few cases reported to date [2–5].

Here we describe a case of AF in a 23-year-old Tharu man; diagnosis followed detailed radiological imaging as well as an immunohistochemistry (IHC) panel. This case report highlights the importance of considering this lesion for any patient presenting with progressive, although painless, trismus with radiological imaging revealing loss of musculoaponeurotic planes within the infratemporal fossa along the pterygoid muscles. IHC panels aid in differentiating it from its malignant counterpart: fibrosarcoma. Our case was also unusual because it involved the infratemporal region, pterygoid muscles, and invasion of the right maxillary sinus.

## Case presentation

A 23-year-old Tharu man from a remote village in Nepal presented to our out-patient clinic with a history of progressive difficulty in opening his mouth for the last 2 months. He had significant weight loss in that period because of the restrictions in taking oral feeds and chewing them. His symptom was not associated with any pain. There was no history of trauma, fever, joint pain, dental pain, rashes, or any other swellings associated with the symptom. His medical history was positive for having been on anti-tubercular therapy (ATT) for suspected abdominal tuberculosis for the last 3 months. There was no significant past surgical or any relevant positive family history. A local examination did not reveal any visible swelling or any features of inflammation around his temporomandibular joint (TMJ). An intra-oral examination was also normal. His mouth opening was of only one finger width. There were no palpable lymph nodes in his body. A general systemic examination was normal.

**Fig. 1:**
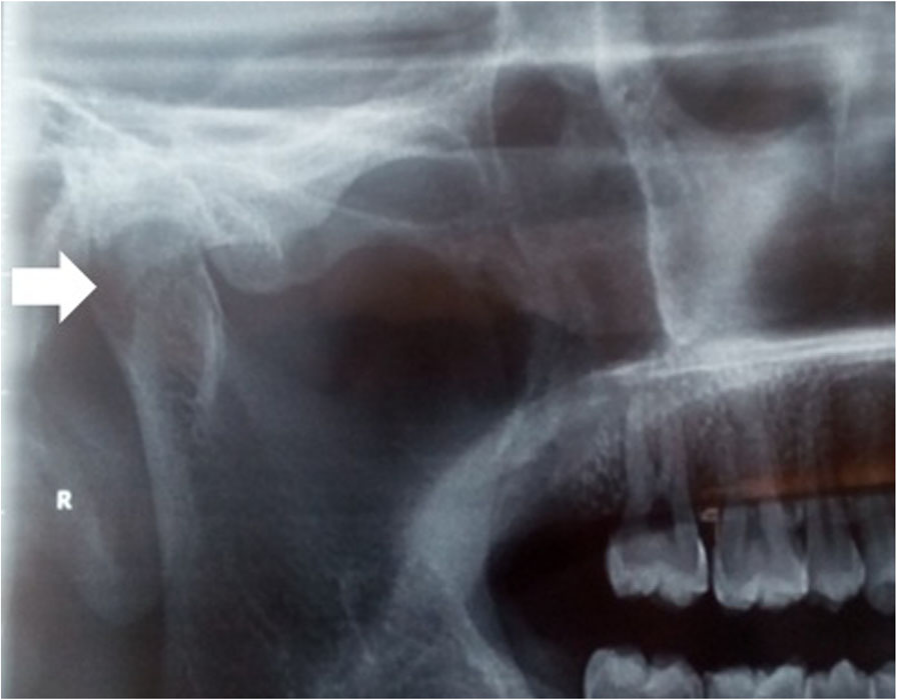
X-ray image showing a normal temporo-mandibular joint (arrow head)

**Fig. 2:**
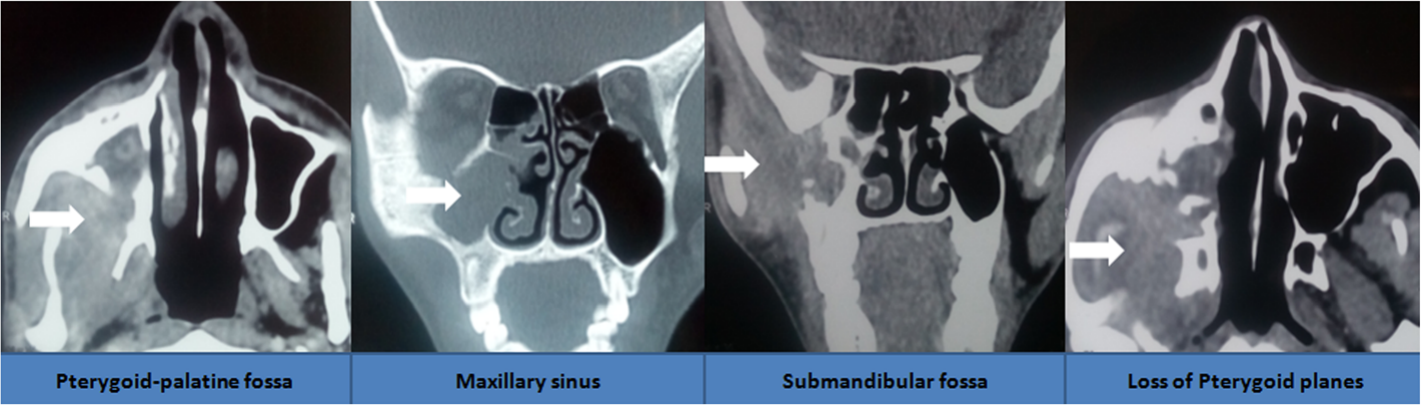
Compute tomography images showing extension of the lesion in the infra-temporal region with loss of pterygoid muscle planes and involvement of the maxillary sinus as well (as shown by arrowheads in respective panels)

**Fig. 3:**
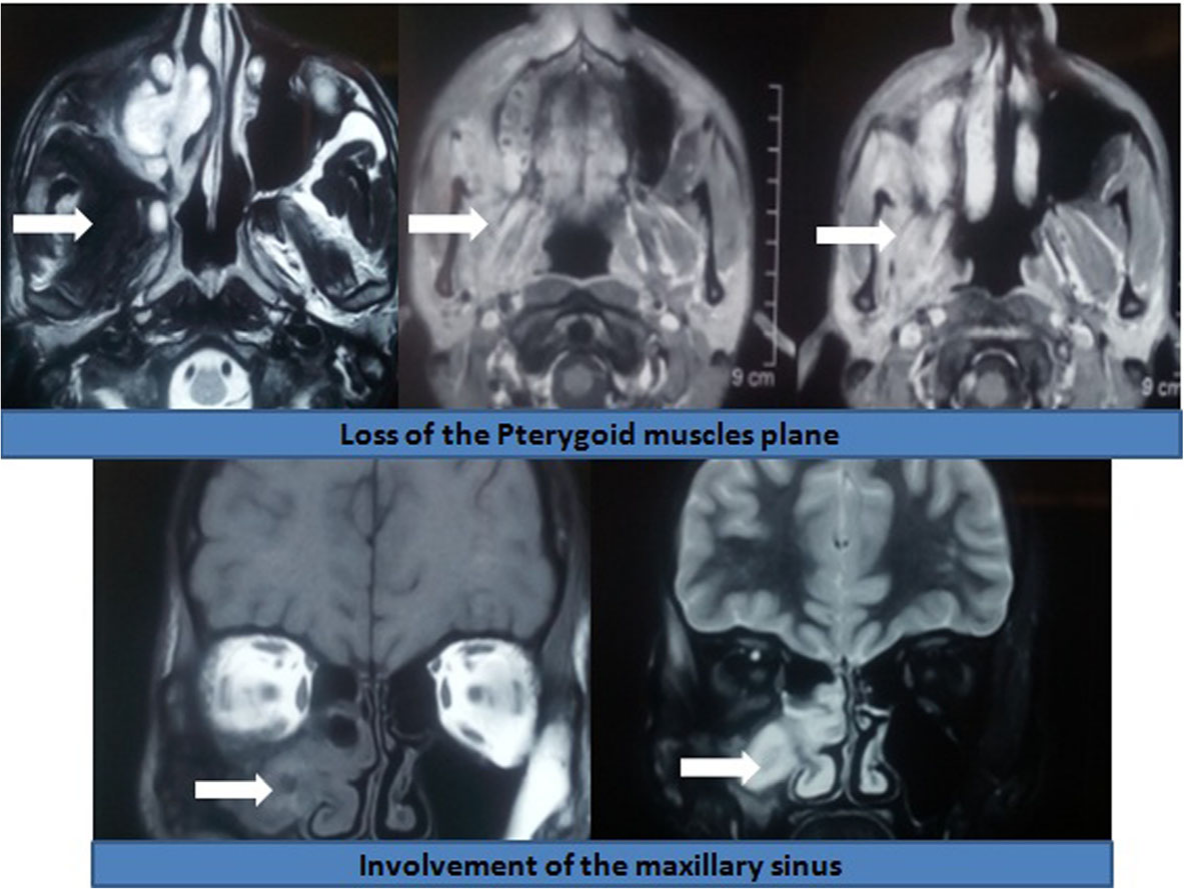
Magnetic resonance images showing the extension of the lesion with characteristic findings in different image sequences (as depicted by arrowheads in respective image panels)

An X-ray of his TMJ was normal (Fig. 1). Computed tomography (CT) and magnetic resonance imaging (MRI) revealed an ill-defined lesion in his right infratemporal region extending to the pterygopalatine fossa and the maxillary sinus (Figs. 2 and 3). There was loss of pterygoid muscle planes. Because of the severity of his symptoms and the imaging findings, an infratemporal approach was undertaken for safe resection and biopsy from the lesion. Our working differential diagnosis was of sarcoma and fibrous variant of tuberculosis. On intraoperative examination, we found an ill-defined fibrous lesion that invaded all the structures in his infratemporal region and insinuated into his maxillary sinus through the pterygopalatine fossa. Safe planes were dissected and biopsy from the lesion was taken.

Postoperatively, his mouth opening improved to three finger width. He also started gaining weight owing to the ease in eating and chewing.

**Fig. 4:**
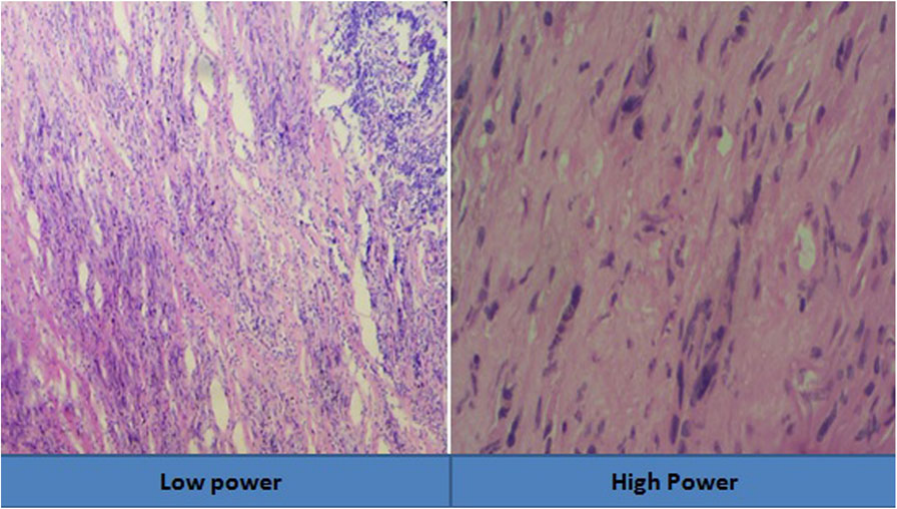
Histology of the lesion at low and high magnification

**Fig. 5:**
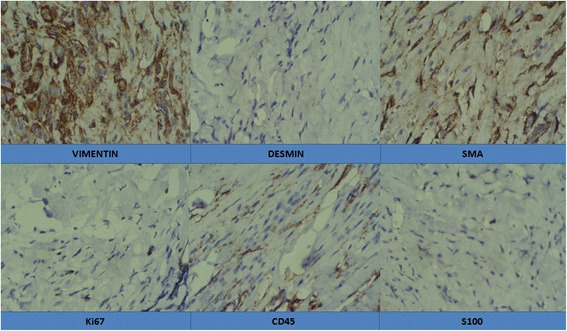
Findings in the immunohistochemistry panel

Histology revealed dense fibrocollagenous tissue with moderate to heavy lymphoplasmacytic infiltrates with entrapped muscle with cells of small nuclei arranged in a linear pattern (Fig. 4). There was no evidence of cellular atypia, mitosis, or necrosis. An IHC panel was positive for vimentin, invariably positive for smooth muscle actin (SMA) but negative for desmin, S-100, and CD45 with Ki-67 value of < 1% (Fig. 5). An acid-fast bacilli (AFB) stain was also negative.

The final diagnosis of AF (desmoid tumor) was eventually made. Our patient had been advised to start weekly methotrexate therapy but he denied it citing their poor economic status.

He has been on regular follow up with no deterioration in his symptoms in the last 4 months.

## Discussion

AF is a rare entity constituting only 0.3% of all tumors with an annual incidence of around 3 to 4 per million of population [1]. Such a lesion affecting the infratemporal region, pterygoid regions, and involving the maxillary sinus is extremely rare [2–5]. This tumor arises from the musculoaponeurotic plane and is locally invasive thereby it is also named an intermediate sarcoma [6]. Fibromatosis in the infratemporal region mostly presents as painless trismus [2].

Radiological divisions into superficial and deep variants have been made [7]. In MRI images, they are usually isointense or mild hyperintense on T1, heterogeneous high signal on T2, and show enhancement following contrast administration [7].

The pivotal issue in such a lesion is to differentiate it from its malignant counterpart of fibrosarcoma, which may be both clinically and radiologically challenging [8]. Another differential could be a reactive fibrosis; however, it characteristically shows areas of focal hemorrhage and hemosiderin deposits [9].

Although surgery is the mainstay of management, safe resection is most often extremely challenging because of the locally invasive nature of AF and its tendency to insinuate into surrounding vital structures. This locally infiltrative growth pattern results in subtotal excision and thereby results in a higher propensity for subsequent recurrence [10]. Intraoperative frozen section may play a role in tumor-free resection margins [11]. However, ill-defined margins often render them completely unresectable and thereby there is a recurrence rate of up to 80% [12].

To minimize recurrence of AF, the role of radiation, especially in recurrent lesions, at a dose of 54 Gray has been advocated [13]. To minimize the side effects of radiation, new advances in the form of MR-guided high intensity ultrasound therapy and external beam therapy have recently been brought into practice [14–16].

There is also the role of pharmacotherapy using both cytotoxic as well as non-cytotoxic agents in managing such a case in a patient-tailored manner [17].

In selective cases, in which the symptoms are not escalating and repeat imaging shows non-progressive lesions, regular follow up can also be justified [18].

Our case was unusual in the sense that there was involvement of the infratemporal fossa with loss of pterygoid muscle planes as well as invasion to the maxillary sinus. Although the lesion was locally invasive, there has been no aggravation in his clinical symptoms in the last 4 months of his follow-up visits.

## Conclusions

Although AF is rare, this case report adds weight to the importance of considering AF in the differential diagnosis of any cases presenting with a painless but progressive trismus. Histology and a detailed IHC assay are the cornerstones in arriving at a diagnosis. There is still a major debate about the optimal therapeutic management of such cases; however, it is prudent to take a tailor-made approach on a case-by-case basis.

## Abbreviations

AF: Aggressive fibromatosis
AFB: Acid-fast bacilli
ATT: Anti-tubercular therapy
CT: Computed tomography
IHC: Immunohistochemistry
MRI: Magnetic resonance imaging
SMA: Smooth muscle actin
TMJ: Temporomandibular joint
